# A Fully Protective Congenital CMV Vaccine Requires Neutralizing Antibodies to Viral Pentamer and gB Glycoprotein Complexes but a pp65 T-Cell Response Is Not Necessary

**DOI:** 10.3390/v13081467

**Published:** 2021-07-27

**Authors:** K. Yeon Choi, Alistair McGregor

**Affiliations:** Health Science Center, Department of Microbial Pathogenesis & Immunology, College of Medicine, Texas A&M University, Bryan, TX 77807-3260, USA; yeonchoi@tamu.edu

**Keywords:** cytomegalovirus, congenital CMV, guinea pig, glycoprotein complex, gB, gH, pentamer complex, neutralizing antibodies, pp65, CMV vaccine

## Abstract

A vaccine against congenital cytomegalovirus infection is a high priority. Guinea pig cytomegalovirus (GPCMV) is the only congenital CMV small animal model. GPCMV encodes essential glycoprotein complexes for virus entry (gB, gH/gL/gO, gM/gN) including a pentamer complex (gH/gL/GP129/GP131/GP133 or PC) for endocytic cell entry. The cohorts for protection against congenital CMV are poorly defined. Neutralizing antibodies to the viral glycoprotein complexes are potentially more important than an immunodominant T-cell response to the pp65 protein. In GPCMV, GP83 (pp65 homolog) is an evasion factor, and the GP83 mutant GPCMV has increased sensitivity to type I interferon. Although GP83 induces a cell-mediated response, a GP83-only-based vaccine strategy has limited efficacy. GPCMV attenuation via GP83 null deletion mutant in glycoprotein PC positive or negative virus was evaluated as live-attenuated vaccine strains (GP83dPC+/PC-). Vaccinated animals induced antibodies to viral glycoprotein complexes, and PC+ vaccinated animals had sterilizing immunity against wtGPCMV challenge. In a pre-conception vaccine (GP83dPC+) study, dams challenged mid-2nd trimester with wtGPCMV had complete protection against congenital CMV infection without detectable virus in pups. An unvaccinated control group had 80% pup transmission rate. Overall, gB and PC antibodies are key for protection against congenital CMV infection, but a response to pp65 is not strictly necessary.

## 1. Introduction

Human cytomegalovirus (HCMV) is a leading cause of congenital disease, resulting in serious symptomatic disease in newborns including impaired vision, mental retardation, sensorineural hearing loss (SNHL), and microcephaly [1,2,3,4]. In the United States, approximately 8000 babies are born each year with disabilities associated with congenital cytomegalovirus infection, and up to 30% of SNHL in children is attributed to congenital CMV [5,6]. Globally, it is estimated that a million babies are born each year with congenital CMV [4], and a vaccine is considered a high priority [7]. However, an effective vaccine has been an elusive goal complicated by the existence of multiple strains of HCMV with the potential for re-infection despite convalescent immunity. Consequently, congenital CMV infection can occur by reactivation of maternal latent infection or by primary infection during pregnancy, as well as secondary viral infection by a new strain in convalescent individuals [8]. HCMV species specificity precludes direct study in animals which require species-specific animal CMV. There are only two models for congenital CMV (guinea pig and rhesus macaques). Currently, no vaccines have been evaluated for protection against congenital CMV infection in the non-human primate model (NHP) model. The guinea pig is the focus of this current study and requires species-specific guinea pig cytomegalovirus (GPCMV). Congenital CMV infection in this model results in disease, including SNHL, in newborn pups [9,10,11,12]. Various intervention strategies against congenital CMV infection have been evaluated in this preclinical model [13,14,15].

In HCMV, viral glycoproteins complexes are required for fibroblast cell entry (gB; gM/gN; gH/gL/gO), and these are neutralizing antibody targets [16,17,18,19,20,21,22,23]. GPCMV encodes homolog glycoprotein complexes to HCMV, which are essential for GPCMV infection of fibroblast cells by direct fusion and are neutralizing target antigens [24,25,26,27,28]. Interestingly, the HCMV gO glycoprotein is highly variable in amino acid sequence in clinical strains compared to other glycoproteins [29]. The significance of this variation is poorly understood but might be linked to cell tropism. A similar divergence of gO amino acid sequence has also been observed between GPCMV strains [30]. Additionally, HCMV clinical strains encode a glycoprotein pentamer complex (gH/gL/UL128/UL130/UL131, or PC) that is necessary for endocytic pathway entry of non-fibroblast cells and is a neutralizing antibody target [23,31]. GPCMV encodes a similar functional pentamer complex (gH/gL/GP129/GP131/GP133), which is necessary for virus infection of a variety of non-fibroblast cell types including epithelial, endothelial cells, macrophage, and placental trophoblasts [24,25,27,32,33,34]. Consequently, the PC is important for GPCMV dissemination in the animal and congenital infection [25,27,33]. Platelet-derived growth factor receptor alpha (PDGFRA), present in fibroblasts, has been identified as the cell receptor necessary for both HCMV and GPCMV direct fusion pathway of entry in fibroblasts requiring gB and gH/gL/gO [27,35,36,37,38]. Several receptors/co-receptors have been identified for PC-dependent cell entry of HCMV [23,39,40,41,42,43,44], but the process of PC-dependent cell entry is only partially understood. The gB glycoprotein is an immunodominant neutralizing target and essential for infection of all cells for both HCMV and GPCMV [24,25,45,46,47]. Consequently, the gB antigen is considered a cornerstone for any vaccine against CMV. Clinical trial studies with a monomeric gB subunit vaccine and MF59 adjuvant have demonstrated partial efficacy [20,48,49]. Various GPCMV monomeric gB vaccine strategies against congenital CMV infection provide at best approximately 50% efficacy [47,50,51]. Vaccine strategies encoding full length gB capable of forming a gB trimer complex found in the virion of HCMV and GPCMV induce higher titer neutralizing antibodies [28,52]. However, this improved gB vaccine strategy is insufficient in the guinea pig model to provide adequate cross strain protection [53]. Further, in both HCMV and GPCMV, gB antibodies are less effective at neutralizing virus on non-fibroblast cells compared to fibroblasts, which might be a basis for reduced gB vaccine efficacy [28,54,55,56].

In addition to neutralizing antibodies, individuals convalescent for HCMV have a robust T-cell response to various viral proteins (including pp65 and IE1), which likely contribute to a protective convalescent immune response [57,58]. GPCMV encodes homologs to these viral proteins [15,24,59,60]. HCMV is a life-long infection, and the burden of the T-cell-mediated immune response, especially directed to the immunodominant T-cell target pp65 viral tegument protein [61], may potentially contribute to immunosenescence long term [62]. Consequently, a vaccine strategy against CMV that induces a potent response to pp65 could unintentionally contribute to future immunosenescence. Additionally, there is a potential risk for the generation of autoantibodies associated with pp65 antigen immune response as demonstrated recently in a mouse model [63]. HCMV pp65 is non-essential on fibroblast [64] but is an important innate immune evasion factor controlling the cGAS/STING antiviral pathway [65,66]. GPCMV GP83 tegument protein is a functional pp65 homolog involved in innate immune evasion by targeting guinea pig IFI16, and cGAS and is an immunodominant T-cell target [26,67,68]. A defective alphavirus-based GP83 vaccine strategy provided limited protection against congenital infection, and a recombinant defective adenovirus GP83 vaccine demonstrated limited cross strain protection [67,68]. Additionally, a recombinant LCMV vaccine strategy based on a combination of gB and GP83 failed to provide significantly higher levels of protection against congenital infection than a gB only vaccine approach [69]. Overall, this suggested that there was potentially a limited benefit of an immune response to pp65 homolog antigen as part of a vaccine against congenital CMV infection in the guinea pig model, especially since there might be a limit for T-cell prevention of diaplacental transmission compared to neutralizing antibodies. However, complete CD4 depletion in a congenital NHP RhCMV model did indicate that a CD4 T-cell response is important in limiting congenital CMV infection [70]. This could additionally include a response to IE1, which has been an effective vaccine candidate in reducing horizontal transmission in the NHP RhCMV model [71] as well as control of B cell response.

A live-attenuated CMV might be an effective vaccine strategy since it mimics natural infection and likely produces a robust long-lasting immune response. Indeed, live-attenuated viral vaccines have been successfully developed against Rubella virus and varicella zoster virus (VZV), which both have the ability to cause congenital disease [72,73]. We recently demonstrated that a non-replication competent GPCMV or defective infectious single cycle (DISC) vaccine approach for GPCMV can effectively prevent congenital CMV infection [36]. However, this recent approach required the use of a specialized complementing cell line. Additionally, a DISC vaccine required three vaccinations to induce a robust immune response, and ideally, a CMV vaccine would only require a single prime boost strategy to ensure that patients complete a vaccine course. Previously, a GPCMV GP83 knockout mutant virus was generated in the backdrop of a lab-adapted GPCMV [59], and mutant virus was explored as a candidate vaccine which had limited efficacy [74]. Originally, the limited vaccine efficacy was thought to be associated with a loss of cell-mediated immune response to GP83. However, in hindsight, the GP83 mutant virus also lacked the ability to express a PC as it did not encode any of the unique PC components (GP129, GP131 and GP133) and was consequently only able to form the viral triplex complex gH/gL/gO [25,28,36,59]. Similar to HCMV, the PC is an important neutralizing target antigen for infection of non-fibroblast cells [25,28,36,59]. Inclusion of neutralizing antibodies to the PC was recently demonstrated to be necessary to provide comprehensive complete protection against GPCMV and congenital infection [26,36]. Potentially, cell-mediated response to GP83 might not be necessary for protection compared to the requirement of an antibody response to gB, gH/gL, and PC viral glycoprotein complexes. We hypothesized that the inclusion of the PC in a live-attenuated GPCMV vaccine strain would likely provide high level protection against congenital CMV infection, similar to a recent DISC vaccine strategy [36]. We further hypothesized that a cell-mediated response to pp65 homolog would not necessarily be required. Consequently, a GP83 knockout mutant would be a suitable basis for a live-attenuated vaccine strategy since HCMV pp65 and GPCMV GP83 have a similar innate immune evasion function but are non-essential for replication. This would also circumvent any potential adverse effects of a vaccine-induced response to pp65 protein.

A GP83 knockout mutant virus was generated on the background of a PC positive virus (GP83dPC+) [68]. An additional GP83 null mutant was generated in the backdrop of a virus lacking full length GP129 (UL128 homolog) and unable to form PC (designated GP83dPC−). This allowed for the evaluation of vaccine immune response in the presence and absence of PC and comparison with viral glycoprotein antibody response in animals convalescent for wild type GPCMV infection. In vaccine protection studies, GP83dPC+ vaccine efficacy was evaluated for protection against wild type virus dissemination in non-pregnant animals and also explored as a preconception vaccine against congenital CMV. The results demonstrated that the inclusion of the PC in this live-attenuated vaccine strategy resulted in high efficacy against wild type virus challenge with sterilizing immunity and full protection of pups against congenital CMV infection.

## 2. Materials and Methods

### 2.1. Cells, Viruses, and Oligonucleotides

GPCMV (strain 22122, ATCC VR682) and second generation GPCMV BAC [75,76] derived viruses were propagated on guinea pig fibroblast lung cells (GPL; ATCC CCL 158) or guinea pig renal epithelial cells (REPI), and virus titrations were carried out on 12-well plates of GPL cells as previously described [36]. Recombinant defective adenovirus (Ad5) vectors encoding individual glycoproteins (gB, gH, gL, GP129, GP131, GP133) were previously described [24,25,28]. All oligonucleotides were synthesized by Sigma-Genosys (The Woodlands, TX, USA).

### 2.2. GP83 Knockout Mutant Construction

The GPCMV sequence was based on the 22,122-strain genome sequence (Genbank accession #AB592928.1). A shuttle vector to remove the majority of the GP83 coding sequence was generated (pGP83dKm) based on an existing GPCMV subgenomic plasmid. The GP83 locus plasmid was digested at unique sites within the *GP83* gene, KpnI (132990) and BglII (131551), to remove the majority of the GP83 coding sequence (codons 7-485 of 566) in the shuttle vector and substitute in place a kanamycin (Km) PCR cassette with flanking KpnI (5′) and BamHI (3′) restriction sites, inserted into the shuttle vector as previously described [24]. This generated the *GP83* knockout shuttle vector pGP83dKm which was used in targeted recombination of the GPCMV BAC to generate a GP83 knockout mutant [68]. The identical strategy was used to generate a GP83 deletion in GPCMV BAC in the background of PC+ and PC− virus.

### 2.3. Generation of Gene Mutant GPCMV BACmids, Analysis and Generation of Virus

An inducible ET recombination system (GeneBridges, Heidelberg, Germany) was introduced into DH10B bacterial cells containing a GPCMV BAC plasmid [75,76]. Mutagenesis of the GPCMV BAC was performed using linearized shuttle vector pGP83dKm as previously described [77]. Isolated mutant GPCMV BAC colonies were characterized by separate EcoR I and Hind III restriction digestions of BAC DNA to verify the accuracy of the predicted genome profile configuration after mutation [75,76]. Insertion of the Km drug resistance cassette into the viral genome introduced a novel Hind III restriction enzyme site at the site of mutation to enable verification of locus modification. The *GP83* knockout was generated on the backdrop of a retrofitted 2nd generation GPCMV BAC, which expressed a missing full length GP129 (UL128 homolog) in an ectopic locus to enable expression of a complete homolog pentameric complex (gH/gL/GP129/GP131/GP133). Similar evaluation was carried out for GP83 knockout in second generation GPCMV BAC that did not encode ectopic copy of GP129 (Figure S1). The insertion of the kanamycin cassette into the *GP83* locus introduced a novel Hind III site in the genomic Hind III “A” fragment (102380-146446). This generated two novel Hind III fragments of 29.9 kb and 13.7 kb (Figure S1). Specific gene modifications were confirmed by comparative PCR analysis between wild type and mutant GPCMV BACs using common flanking primers for each gene and DNA sequencing of modified locus [25]. GP83 locus was amplified with primers F409 5′CATCAAGATGGTCAACAGGTCGCACGAC and R409 5′TGTCGTAGAGCACTTCGAACCTGACTCTG. 

### 2.4. Generation of Recombinant Virus

For generation of recombinant viruses, large-scale maxi prep GPCMV BAC DNA was transfected onto GPL cells in six-well dishes [78]. The BAC plasmid was also excised from the viral genome via CRE/loxP recombinase strategy [75]. Two independent GP83 mutant GPCMV BAC clones were separately transfected onto GPL cells for each mutant background (PC+ or PC−). A virus stock was generated based on one clone, and mutant virus was designated GP83dPC+ and GP83dPC−. GP83 rescue virus to restore GP83 function was previously described [68].

### 2.5. Ethics

Guinea pig (Hartley) animal studies were carried out under IACUC permit (Texas A&M University, College Station, TX, USA). All study procedures were carried out in strict accordance with the recommendations in the “Guide for the Care and Use of Laboratory Animals of the National Institutes of Health.” Animals were observed daily by trained animal care staff, and animals that required care were referred to the attending veterinarian for immediate care or euthanasia. Terminal euthanasia was carried out by lethal CO_2_ overdose followed by cervical dislocation in accordance with IACUC protocol and NIH guidelines. Animals purchased from Charles River Laboratories were verified as seronegative for GPCMV by toe nail clip bleed and anti-GPCMV ELISA of sera as previously described [24].

### 2.6. GPCMV Vaccine Protection Studies

#### 2.6.1. GP83dPC+ Vaccine Protection Study (Pathogenicity)

Seronegative female guinea pigs (*n* = 12) were vaccinated subcutaneously with the GP83dPC+ twice 25 days apart. At 2 months post last vaccination, animals were challenged with wild type GPCMV (10^5^ pfu, SQ), and animals were evaluated for wild type virus spread following strategy described above for pathogenicity, with animals euthanized (*n* = 3) at various time points (4, 8, 12, and 27 dpi) and target organs and blood evaluated for viral load. A control seronegative unvaccinated group of animals was similarly evaluated for wild type GPCMV dissemination. Each sample collected was evaluated in triplicated minimum of 2 separate assay runs.

#### 2.6.2. GP83dPC+ Preconception Vaccine Protection Study

Seronegative female guinea pigs were randomly assigned to two different groups. Group 1 (GP83dPC+ vaccinated group; *n* = 12) was vaccinated SQ with the GP83dPC+ vaccine and boosted once with a repeat dose. Animals were confirmed for GPCMV seroconversion by an anti-GPCMV ELISA. The immune response to individual glycoprotein complexes was also determined for vaccinated animals prior to mating. Next, the dams were paired with seronegative males for mating. The dams were confirmed to be pregnant by palpation at approximately days 20 to 25 of gestation. A second control group of unvaccinated seronegative females (*n* = 14) was also paired for mating. At the late second trimester/early third trimester, pregnant animals in both groups were challenged with a salivary gland stock of wild type GPCMV (10^5^ PFU) SQ, and the animals were allowed to go to term. The viral load in the target organs (liver, lung, spleen, brain) of live-born or still-born pups was evaluated by real-time PCR.

#### 2.6.3. Pathogenicity Study (GP83dPC−)

GP83 mutant virus (GP83dPC−) was evaluated for pathogenicity in the animal model compared to parental GPCMV(PC−), derived from second generation of GPCMV BAC and virus restored for PC by ectopic expression of GP129 in GP25-GP26 intergenic locus (designated GP129FRT) [25]. Pathogenicity studies were carried out as previously described. GPCMV seronegative animals were randomly assigned into 3 groups of *n* = 12 per group. At day 0, animals were injected (SQ) with 10^5^ pfu of GP83dPC-, GPCMV(PC−), or GP129FRT. At 4, 8, 12, and 27 days post infection (dpi), three animals per group were euthanized, and viral load in target organs (liver, lung, spleen, and blood) was evaluated by real-time PCR assay. Additionally, at 27 dpi, salivary gland tissue was also evaluated for viral load. Each sample collected was evaluated in triplicated minimum of 2 separate assay runs.

### 2.7. Real-Time PCR Assay

Samples (tissues and blood) were collected from euthanized guinea pigs to determine the viral load as previously described. For tissue DNA extraction, FastPrep 24 (MP Biomedical, Irvine, CA, USA) was used to homogenize tissues as a 10% weight/volume homogenate in Lysing Matrix D (MP Biomedicals). Whole blood was collected into ACD anti-coagulant tubes. DNA was extracted using the QIAcube HT (Qiagen, Germantown, MD, USA) according to manufacturer’s tissue or whole blood protocols. Viral load was determined by real-time PCR on a LightCycler 480 (Roche Applied Science) using GPCMV GP44 primers and hydrolysis probe as previously described [25,26]. Data were collected by “single” acquisition during the extension step and with the LightCycler Data Analysis Software (Version 1.5.1; Roche Life Sciences, Santa Clara, CA, USA). Standard curve was generated using serial dilutions of GPCMV GP44 plasmid [78] at known concentrations for quantification and assay sensitivity. The sensitivity of the assay was determined to be 5 copies/reaction. Viral load was expressed as genome copies/mg tissue or ml of blood. Results calculated were a mean value of triplicate PCR runs per sample.

### 2.8. Anti-GPCMV and Individual Glycoprotein Complex Antibody ELISAs

Anti-GPCMV ELISA was carried out as previously described [24]. MaxiSorp ELISA plates were coated with appropriate antigens overnight before being blocked with 2% non-fat milk. Diluted sera starting at 1:80 were added for one hour followed by anti-guinea pig IgG-peroxidase secondary (1:2000). Colorimetric detection was obtained by TMB. Net OD (absorbance 450 nm) was attained by subtracting the OD of Ag− from the OD of Ag+. ELISA reactivity was considered positive if the net OD was greater than or equal to 0.2 as determined by GPCMV negative serum. Individual glycoprotein complex ELISAs were carried out as previously described [36]. All samples were run in triplicates in minimum of three separate assays. ELISA titer was determined as the reciprocal of the highest serum dilution with net OD ≥ 0.2 abs.

#### 2.8.1. Antibody Avidity Assay

IgG avidity was determined following the ELISA protocol with the addition of 6M urea as the dissociating agent as previously described [36]. Briefly, after incubation with diluted test sera, one set of wells was washed with regular wash buffer, whereas the other set of wells was washed with wash buffer containing urea. The ELISA was completed as described above. Results were expressed as an avidity index (AI) in percentage and calculated as AI = (OD of urea washed well/OD regular well) × 100.

#### 2.8.2. Antibody Depletion from Sera

Immunodepletion of antibodies to gB, gH/gL, or PC glycoprotein complex was carried out as previously described [36]. Glycoprotein-depleted sera were used for ELISAs and neutralization assays as described in other sections. The starting serum dilution of 1:80 was adjusted for the 1:2 dilution during the immunodepletion step. Therefore, to reach the starting dilution of 1:80, the depleted serum starting dilution was 1:40.

#### 2.8.3. GPCMV Serum Neutralization

GPCMV neutralization assays (NA_50_) were performed on GPL fibroblast or renal epithelial with wtGPCMV virus stocks. Neutralization assays were carried out as previously described with individual animal serum or pooled sera from convalescent wild type infected animals [36]. Sera was not heat inactivated and supplemented with rabbit complement [28]. Final neutralizing antibody titer was the inverse of the highest dilution producing 50% or greater reduction in plaques compared to virus only control. Neutralization assays were performed from each sample three times. Neutralization assays were performed concurrently with the same virus stocks between groups.

### 2.9. Statistical Analysis

All statistical analyses were conducted with GraphPad Prism (version 7) software. Replicate means were analyzed using Student’s *t*-test (unpaired), with significance taken as a *p* value of <0.05 or as specified in the figure legends. Significance in pup outcome and congenital infection rate was determined using Fisher’s exact test with a *p* value stated in the table legends.

## 3. Results

### 3.1. Generation GP83 Knockout GPCMV Vaccine Strain on Backdrop of PC+/PC− Virus

**Figure 1:**
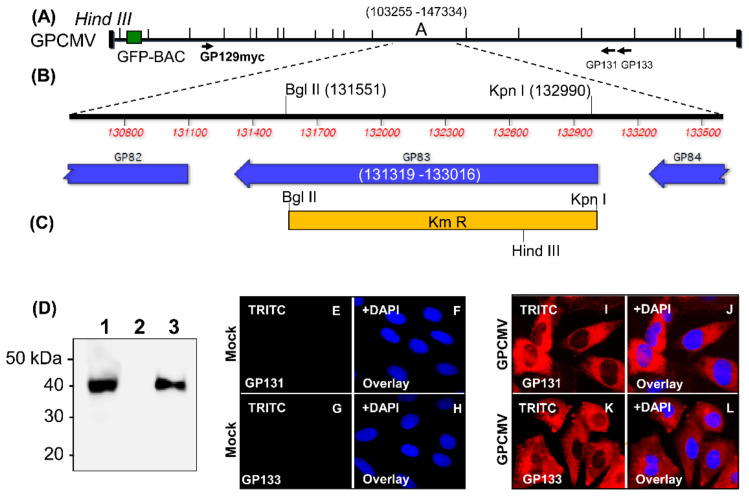
Generation of GP83 null PC+ GPCMV mutant virus (GP83dPC+). (**A**) Linear diagram of the GPCMV genome showing Hind III genomic map, location of excisable BAC plasmid (GFP-BAC), and ectopic GP129 (Gp129myc) in GP25-GP26 intergenic locus to enable PC expression. Location of additional unique PC components GP131 and GP133 also indicated. (**B**) GP83 locus encoded in Hind III ‘A’ GPCMV subgenomic fragment (nucleotides 103255–147334) and location of restriction enzyme sites Bgl II (131551) and Kpn I (132990) within GP83 coding sequence (131319–133016) used to delete majority of ORF via shuttle vector pGP83dKm and subsequently modify the GPCMV BAC via ET recombination to remove the majority of the GP83 coding sequence (codons 7–485) and substitute in place a kanamycin (Km) cassette (**C**). GP83 mutant virus (GP83dPC+) generated by transfection of GPCMV BAC DNA onto GPL fibroblast cells and excision of BAC plasmid. Virus infected cell expression of ectopic GP129 verified by Western blot (**D**) of GP83dPC+ infected cell lysate (lane 1), mock infected GPL (lane 2), and parental (GP83+) virus GP129FRT (lane 3) for myc epitope tagged GP129 protein as described in Materials and Methods. Expression of the additional unique components of the PC (GP131 and GP133) in GP83dPC+ infected GPL cells was verified by immunofluorescence assay with custom antibodies to GP131 (**I**,**J**) and GP133 (**K**,**L**). Primary antibodies: mouse anti-GP131 or anti-GP133 (Genscript); and secondary: anti-mouse IgG-TRITC conjugate (Sigma). Control immunofluorescence assays on mock infected cells for GP131: (**E**,**F**); and GP133: (**G**,**H**). Counterstain DAPI and TRITC overlay for mock and virus infected cells: (**F**,**H**,**J**,**L**). Images at 20× magnification.

*GP83* null mutant viruses were generated to remove the majority of the GP83 coding sequence (codons 7-485 of 566 coding sequence) via GPCMV BAC mutagenesis. Two versions of the *GP83* knockout mutant were made: (1) GP83dPC+ with virus background PC+ (Figure 1) [68]; (2) GP83dPC− with virus background PC− (Figure S1). The PC+ *GP83* null mutant was generated on the backdrop of a viral genome retrofitted to encode the PC by ectopic expression of missing full length UL128 homolog (GP129) in the *GP25–GP26* intergenic locus. Ectopic GP129 in virus positive for GP83 restored virus tropism and pathogenicity in vivo, including congenital CMV in animals [25,27,33]. The generation of a *GP83* mutant virus (GP83dPC+) was recently described, and mutant virus was attenuated in vivo despite encoding PC [68]. The expression of unique components of the PC (GP129, GP131, and GP133) by GP83dPC+ mutant virus was verified by Western blot of infected cell lysate (ectopic GP129 myc tagged protein, Figure 1D) or immunofluorescence of GP83dPC+ virus infected GPL fibroblast cells with custom antibodies (Genscript) [25] for GP131 (Figure 1I,J) and GP133 (Figure 1K,L). An additional *GP83* null mutant was created in the background of PC− virus via mutagenesis of the second generation GPCMV BAC lacking full length GP129 [25,75] and virus designated as GP83dPC− (Figure S1, Supplementary Material). Both GP83 mutant viruses were compared for pathogenicity in animals (Figure S2, Supplementary Material) and evaluated for an immune response to viral glycoprotein complexes plus neutralizing antibody titers in separate groups of vaccinated animals. However, GP83dPC−was not used in vaccine protection studies since a previous DISC vaccine had shown the necessity for an immune response to PC for enhanced vaccine efficacy.

A recent publication from our laboratory more fully defined the phenotype of the GP83dPC+ mutant virus, which had normal growth compared to wild type virus on fibroblast cells but had substantially increased sensitivity to IFN-I compared to wild type virus [68]. GP83dPC− had a similar phenotype to GP83dPC+ on fibroblasts (data not shown). Additionally, the GP83dPC+ virus was attenuated on non-fibroblast cells and impaired for virus dissemination in animals, despite encoding PC, which was linked to the GP83 innate immune evasion function in the IFI16/cGAS/STING antiviral pathway [68]. Importantly, there was no detectable GP83dPC+ virus in the salivary glands of infected animals, which is considered a site for persistent long term virus replication [68] (Figure S2). Not unexpectedly, GP83dPC− was further attenuated in animals compared to GP83dPC+ virus because of the lack of PC for non-fibroblast infection (Figure S1, Supplementary Material) [25,27,33,68]. Overall, both the GP83dPC+ and GP83dPC− mutant viruses were considered sufficiently attenuated to be evaluated as a candidate vaccines.

### 3.2. Evaluation of Immune Response of GP83dPC+ in Vaccinated Animals

**Figure 2:**
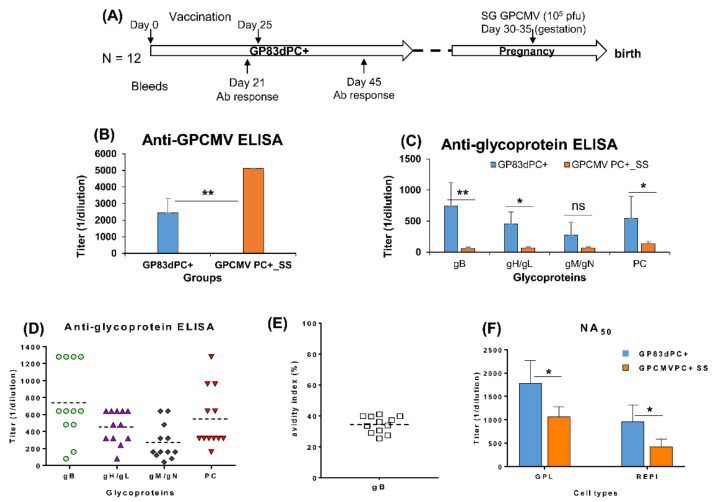
GP83dPC+ vaccination schedule and immune response. (**A**) Overview of GP83dPC+ preconception vaccine and challenge schedule. Animals were vaccinated 2 times (10^5^ pfu) via SQ route: day 0 and 25. Serum collected 3 weeks post each vaccination (days 21 and 45). Dams were mated, and during late second trimester of pregnancy, animals were challenged with 10^5^ pfu salivary gland (SG) stock wt GPCMV (SQ), then followed to term. (**B**) Mean anti-GPCMV ELISA titer from sera collected at D45 in animals inoculated with GP83dPC+ (blue) or sera from single inoculation of wtGPCMV (orange). (**C**) Mean anti-glycoprotein complex ELISA titers (gB, gH/gL, gM/gN, PC) from sera collected at D45 in animals inoculated with GP83dPC+ (blue) or sera from single inoculation of wtGPCMV (orange). (**D**) Evaluation of GP83dPC+ vaccine glycoprotein complex ELISA titers for sera from individual animals (collected at D45). Specific anti-glycoprotein complex immune responses: anti-gB (circle); anti-gH/gL (triangle); anti-gM/gN (diamond); anti-PC (upside down triangle). (**E**) GPCMV gB avidity index for individual animal sera. (**F**) Neutralization of GP83dPC+ vaccine sera or wt GPCMV PC+ (SS) pooled convalescent sera on GPL or REPI cells. Mean ELISA and neutralization values are a result of assay triplicates with each sample run a minimum of three independent times. Statistical analysis determined by Student’s *t*-test. * *p* < 0.05; ** *p* < 0.005; ns = non-significant.

Seronegative dams were vaccinated (10^5^ pfu, SQ) with the GP83dPC+ at two independent time points (0 and 25 days), and animals were bled at days 21 and 45 post initial injection for evaluation of immune response (Figure 2A). Subsequently, after the characterization of immune response, dams were used in a congenital CMV vaccine protection study. The antibody ELISA titer results for anti-GPCMV (Figure 2B) and individual glycoprotein complexes (gB, gH/gL, gM/gN and PC), Figure 2C, are shown for overall mean values at day 45 time point bleeds and are compared to the pooled sera from animals convalescent for wild type virus after a single dose of GPCMV (10^5^ pfu, SQ) [30]. The anti-GPCMV ELISA titer for GP83dPC+ vaccinated animals was significantly lower than wild type GPCMV infected animals (Figure 2B, *p* < 0.005). However, GP83dPC+ animals induced a higher antibody immune response to all glycoprotein complexes compared to wild type infected animal sera (Figure 2C) determined by ELISA, and this was statistically significant except for the gM/gN titer, which evoked the lowest response for both groups. The immunodominant gB evoked the highest antibody titer to a glycoprotein complex in GP83PC+ vaccinated animals (Figure 2C).

The evaluation of individual animal antibody responses to viral glycoprotein complexes in the GP83dPC+ vaccinated animals (Figure 2D) revealed a broad range in anti-gB titers. The immune responses to gH/gL were more similar, while the response to gM/gN was more limited. The immune response to the PC was the most variable between animals (160 to 1280 titer range). An evaluation of antibody avidity for gB between GP83dPC+ vaccinated animals indicated a more similar avidity value despite a wide spectrum in anti-gB titers between animals (Figure 2D,E). A similar anti-gB avidity result was also observed in a previous GPCMV DISC vaccine strategy [36]. Neutralization assays (NA_50_) for pooled GP83dPC+ vaccinated animal sera on fibroblast and renal epithelial cells (Figure 2F) were compared to wild type virus convalescent pooled sera (GPCMVPC+ SS). The results demonstrated that GP83dPC+ vaccinated animals were able to neutralize virus on both fibroblast and epithelial cells approximately twofold more effectively than wild type GPCMV convalescent sera (Figure 2F). However, NA_50_ epithelial titers for both sera were approximately two-fold lower compared to their NA_50_ fibroblast titer (Figure 2F).

### 3.3. Comparison of Immune Responses between GP83dPC+ and GP83dPC− Vaccinated Animals

**Figure 3:**
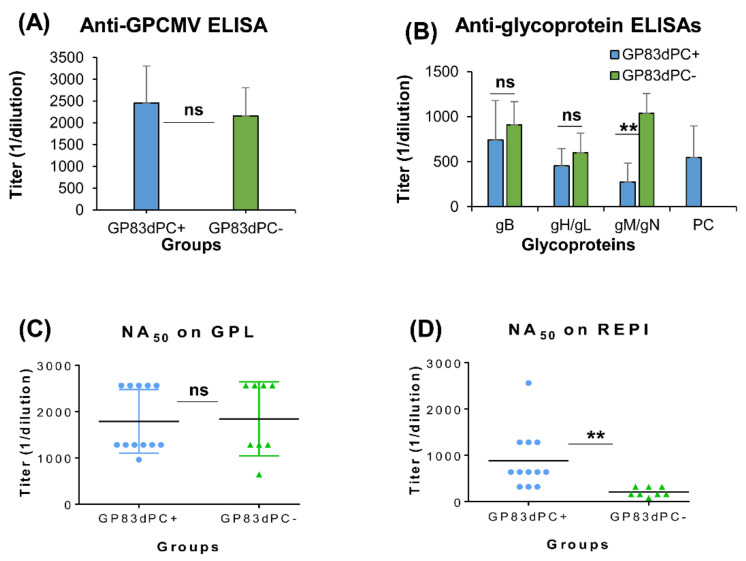
Comparative immune response of animals inoculated with GP83dPC+ vs. GP83dPC− vaccine. (**A**) Mean anti-GPCMV titer comparison of D45 sera from animals inoculated with GP83dPC+ (*n* = 12; blue) or GP83dPC− (*n* = 8; green). (**B**) Comparative mean anti-glycoprotein complex ELISA titers (gB, gH/gL, gM/gN, PC) from sera in animals inoculated with GP83dPC+ (blue) or GP83dPC− vaccine (green). Comparative GPCMV neutralization (NA_50_) on GPL cells (**C**) or REPI epithelial cells (**D**) of individual serum from vaccinated animals from both groups: GP83dPC+ vaccinated (blue); GP83dPC− vaccinated (green). Black solid line represents mean titer. Mean ELISA and neutralization values are a result of assay triplicates with each sample run minimum of three independent times. Statistical analysis determined by Student *t* test; ** *p* < 0.005; ns = non-significant.

A second GP83 null mutant generated on the backdrop of virus lacking the PC (GP83dPC−) was used to vaccinate seronegative animals (*n* = 8) with an identical vaccine regime to the GP83dPC+ virus (Figure 2A). GP83dPC− vaccination evoked similar anti-GPCMV titers in animals to those of GP83dPC+ vaccinated animals (Figure 3A). Overall, the immune response to gB, gH/gL was similar between groups (Figure 3B). However, sera from the GP83dPC− group of animals had a higher ELISA titer for gM/gN complex (Figure 3B), and this difference was previously observed between GPCMV DISC PC+ and PC− vaccinated animals [26,36]. Comparative evaluations for sera from individual GP83dPC+ and GP83dPC− vaccinated animals were carried out by wild type GPCMV neutralization assays to establish 50% inhibition titers (NA_50_). On fibroblast (GPL) cells, NA_50_ values were roughly similar between vaccine groups (Figure 3C). However, on epithelial (REPI) cells, GP83dPC+ vaccinated animals had a statistically higher NA_50_ titer compared to GP83dPC− vaccinated animals (Figure 3D; *p* < 0.005). A statistical difference between groups remained after the exclusion of the one animal in the GP83dPC+ group with the highest NA_50_ titer on epithelial cells. We concluded that the difference in neutralizing titers between groups was based on GP83dPC− not encoding the PC and consequently being unable to induce a specific antibody response to PC outside of gH/gL, which resulted in a lower NA_50_ titer on epithelial cells compared to GP83dPC+ vaccinated animals.

### 3.4. Depletion of Antibodies to Specific Viral Glycoprotein Complexes Demonstrates Similar Results between GP83dPC+ and DISC (PC+) GPCMV Vaccine Sera

**Figure 4:**
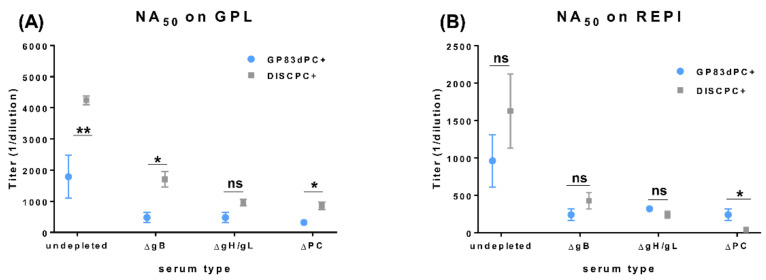
DISC(PC+) vaccine vs. GP83dPC+ vaccine for GPCMV neutralization by complete or sera depleted of antibodies to specific viral glycoprotein complexes (gB or gH/gL and PC). Neutralizing antibody titers (NA_50_) on different cell types of pre- and post-glycoprotein-complex antibody depleted vaccine sera. Pooled sera from GP83dPC+ vaccinated animal group or historical DISC(PC+) pooled vaccine sera [36] were tested for NA_50_ against wt GPCMV (PC+) on GPL fibroblast or REPI epithelial cells. (**A**) Antibody neutralization of wt GPCMV (PC+) using pre- (native) or post-gB (ΔgB)-, post-gH/gL (ΔgH/gL)-, or post-PC (ΔPC)-depleted sera on the GPL cell line. (**B**) Antibody neutralization of GPCMV with pre- or post-depleted sera tested on the REPI cell line. * *p* < 0.05; ** *p* < 0.005; ns = non-significant, Student’s *t* test.

We previously reported the development of a conditional non-replication competent DISC GPCMV vaccine strategy [26,36]. Both PC+ and PC− DISC vaccine strategies had been evaluated for an ability to induce a protective response in vaccinated animals against subsequent challenge during pregnancy with wild type GPCMV. However, only the DISC vaccine encoding the PC was able to provide complete protection against congenital CMV with sera inducing a specific antibody response to the PC and enhanced virus neutralization on non-fibroblast cells compared to DISC(PC−) vaccine sera [36]. Using historical pooled sera from DISC(PC+) vaccinated animals [36], a comparison was made with pooled sera from GP83dPC+ vaccinated animal pooled sera for virus neutralization on fibroblast or epithelial cells. A previously established protocol utilized for selective separate depletion of GPCMV gB, gH/gL, or PC antibodies from sera [36] and vaccine sera was used to enable side by side comparison of GPCMV neutralization by DISC(PC+) and GP83dPC+ sera undepleted (native) or depleted of antibodies to specific viral glycoprotein complexes. The depletion of anti-gB, anti-gH/gL, or anti-PC was verified by specific glycoprotein complex antibody ELISA pre- and post-sera depletion stages (data not shown; [36]). The results show a similar pattern of NA_50_ for both DISC(PC+) and GP83dPC+ vaccine sera. Prior to depletion, both vaccine sera have the highest NA_50_ titers on fibroblasts compared to epithelial cells. The depletion of anti-gB had an impact on both fibroblast and epithelial NA_50_ titers, which demonstrated the importance of gB for infection of both cell types as previously noted [28] (Figure 4). Additionally, anti-gH/gL and anti-PC depletion reduced NA_50_ values on both cell types, which would be expected since gH/gL is present as part of the PC or trimer complex where both are important for cell free virus entry, and PC is important specifically for the endocytic pathway of entry (Figure 4) [25,28,37]. Overall, the results demonstrate that antibody response to gB, gH/gL, and PC is important for effective neutralization of virus infection. It is likely that there is a cross enhancement of neutralizing antibody effect associated with anti-gB together with anti-gH/gL with an ability to block the fusogenic step required for cell entry, which occurs in HCMV, and a similar interaction demonstrated for GPCMV [37].

### 3.5. GP83dPC+ Vaccinated Animals Are Protected against Wild Type Virus Challenge

**Figure 5:**
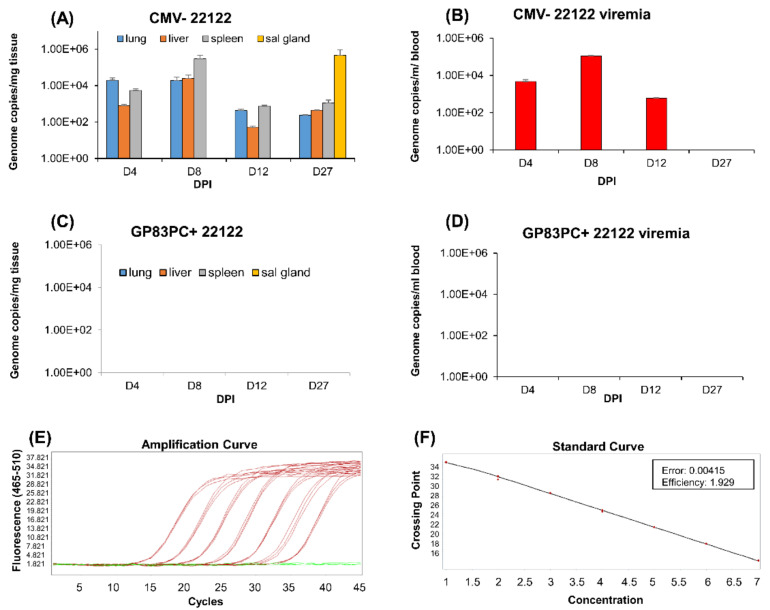
Comparative dissemination of GPCMV to target organs in seronegative and GP83dPC+ vaccinated animals. Seronegative animals, (**A**,**B**), and GP83dPC+seropositive animals, (**C**,**D**) (*n* = 12 per group), were challenged with wt GPCMV (10^5^ PFU). At various days (4, 8, 12, and 27 dpi), 3 animals per group were evaluated for the viral load in the target organs by real-time PCR of DNA extracted from tissues, (**A**,**C**). Mean viral load is plotted as the number of viral genome copies/mg tissue. Salivary gland (sal gland) tissue was evaluated only at day 27. Mean viremia at 4, 8, 12, and 27 dpi plotted as the number of genome copies/mL blood (**B**,**D**). The target organs were lung, liver, spleen, salivary gland, and blood. Experiments carried out in triplicates in minimum of two independent assay runs. Representation of real-time PCR standard amplification, (**E**,**F**). A known concentration of GPCMV *GP44* plasmid DNA was diluted 10-fold and run in triplicate, as stated in Materials and Methods, to generate the amplification curve (**E**) and the standard curve (**F**). The red lines indicate positive amplification; the green lines indicate the negative no-template control.

Previous GPCMV DISC vaccine studies had indicated that sterilizing immunity was possible against wild type GPCMV (22122) virus challenge [36]. Consequently, we evaluated protection against wild type GPCMV (22122 strain) in GP83dPC+ vaccinated non-pregnant female animals. A group of seronegative animals (*n* = 12) was vaccinated under identical vaccine regime as described in Figure 2A and verified for seroconversion by anti-GPCMV ELISA. At 2 months post last vaccination, animals were challenged with wild type salivary gland stock GPCMV (10^5^ pfu, SQ). A control group of seronegative animals (*n* = 12) was similarly challenged with wild type virus. At various days post infection (4, 8, 12, and 27 dpi), animals were euthanized (*n* = 3/group), and viral load in target organs and blood was determined by real-time PCR of extracted DNA. Figure 5 shows viral load in tissues (Figure 5A) and blood (Figure 5B) in control seronegative unvaccinated animals, which showed challenge virus dissemination to the target organs lung, liver, and spleen (4–27 dpi) and blood (4–12 dpi). Virus was also detected in the salivary gland at 27 dpi in the control group. In comparison, the vaccinated animals did not have detectable viral load in target organs (Figure 5C) or blood (Figure 5D) at any day post infection. Overall, the results indicated that the immune response to GP83dPC+ was effective at preventing challenge wild type virus dissemination. Potentially, low level challenge virus was present in tissue or blood in the vaccinated animals but below the level of detection of our real-time PCR assay (Figure 5E,F).

### 3.6. Vaccinated Animals Are Protected against Congenital CMV Infection

GP83dPC+ vaccinated dams (*n* = 12), Figure 2A, were mated with seronegative males. At approximately 30–35 days of pregnancy (2nd trimester), dams were challenged with wild type salivary gland stock virus (10^5^ pfu, SQ). A control group of seronegative unvaccinated animals (*n* = 14) were mated and similarly challenged with wild type virus during 2nd trimester. Animals were allowed to proceed to term, and pups from both groups were evaluated for viral load. A summary table of the congenital CMV study outcome is shown in Table 1, which compares vaccine group litters of pups to those from unvaccinated control group. In the control unvaccinated group, the number of still-born pups was almost as high as live-born pups (Table 1), and all still-born pups in the non-vaccinated group were positive for virus (Table 2). Although a small number of still-births occurred in the vaccine group, this number was small (3) compared to live pups (34), and no litter had only still-born pups, which occurred in the unvaccinated control group (4 litters), Table 1. Additionally, one dam in the vaccine group was euthanized pre-term at 18 days post wild type virus challenge due to unknown health issues where 4 pups were harvested and included in the study. These still-born pups were negative for viral load as were all pups in the vaccine group (Table 2). Still-born pups in the vaccine group were assumed not to be associated with congenital CMV infection as virus was not present and pup death was associated with a complication of pregnancy. However, we cannot rule out the possibility of virus being present in still-born pups of the vaccine group at below detectable assay levels. Overall, the GP83dPC+ vaccine strategy was highly successful with a reduction of congenital GPCMV transmission rate from 80% (unvaccinated group) to 0% (GP83dPC+ vaccine group). We concluded that as with an earlier DISC vaccine strategy, the inclusion of the PC antigen enhances vaccine efficacy [26,36]. Additionally, a T-cell response to pp65 homolog was not necessary for vaccine protection against congenital infection by prototype 22122 strain GPCMV. However, it is possible that the GP83dPC+ virus did evoke a T-cell response to IE1 protein [60] which contributed to protection, but this awaits future study.

## 4. Discussion

A protective vaccine strategy against congenital CMV infection is a high priority but an elusive goal. An effective vaccine needs to exceed the levels of immunity achieved in convalescent natural CMV infection since congenital infection can occur by primary or secondary CMV infection. Importantly, convalescent immunity is limited in its ability to prevent infection by a new strain. The immunological cohorts for complete protection against congenital CMV infection are poorly defined, both in the context of HCMV and animal models. However, the antibody response to viral antigens, including neutralizing antibodies to the viral glycoprotein complexes, is considered highly important. Potentially, a vaccine strategy might have to attain sterilizing immunity against CMV to provide the highest level of protection, and this was achieved with the GP83dPC+ vaccine strategy. However, our current studies were limited to the 22,122 prototype strain GPCMV challenge, and the vaccine strategy may potentially have more limited efficacy against cross strain protection, but this awaits future evaluation.

Low antibody titers, or non-neutralizing antibodies, to viral glycoprotein complexes might be a significant risk factor for congenital CMV, and the GP83dPC+ vaccine induced higher titers to viral glycoprotein complexes compared to convalescent animals, but there is likely room for improvement. A significant focus for CMV vaccines has been devoted to the immunodominant gB protein. Previous gB vaccine studies in the guinea pig model have utilized truncated gB with an inability to form higher order multimer gB trimeric complex and neutralizing target antigen, which in part limits vaccine efficacy against congenital CMV [47,50,51]. The expression of a full-length GPCMV gB antigen, rather than a truncated gB in a recombinant defective adenovirus vector vaccine strategy (AdgB), induced a more potent neutralizing antibody response by forming gB trimer antigen in addition to gB monomer antigens [28]. Recombinant GPCMV expresses the full length gB with an ability to form trimeric gB, as well as a potential for interaction with gH/gL [37,79]. However, despite the essential requirement for gB on all cell types, the neutralizing antibodies were less effective on non-fibroblast cells, including renal epithelial, trophoblasts, and amniotic sac cells [27,28]. Consequently, the AdgB vaccine strategy reduced but did not fully prevent wild type virus dissemination in vaccinated animals [28]. The results indicated that additional target antigens are necessary to induce an effective protective response. The viral glycoprotein complexes gH/gL and gM/gN are essential in GPCMV, and antibodies to these complexes, in addition to gB, contribute to virus neutralization [24]. However, on cell types that are dependent upon the PC for virus entry, an additional antibody response to the PC is required to effectively neutralize virus infection [27,28,36]. In HCMV, neutralizing antibodies to the PC have a broad range of efficacy against various clinical strains. A cross strain protection study has recently been evaluated for GPCMV where a full length gB vaccine strategy against a novel clinical GPCMV strain demonstrated poor efficacy, and additional studies demonstrate the importance of the immune response to PC for broad protection against GPCMV strains similar to HCMV [30,53].

In this current study, we demonstrate that the inclusion of the PC in a live-attenuated GPCMV vaccine strain induces better virus neutralization on epithelial cells compared to a PC-negative vaccine strain (GP83dPC−). Since GP83dPC− virus expressed full-length GP131 and GP133, we also concluded that a detectable antibody response to individual unique PC components, GP131 and GP133 (data not shown), is non-neutralizing, and PC neutralizing antibodies are directed to the complete PC or gH/gL. The GP83dPC+ vaccine strain was able to induce a protective immune response with a prime and single boost approach. In contrast, a previous DISC GPCMV(PC+) vaccine required a prime and double boost strategy to induce protective immunity [26,36]. This might be a significant factor for vaccination with less injections being more desirable, but this might be at the cost of a lower anti-PC titer. An advantage of the pp65 null viral strain (both HCMV and GPCMV) is that the mutant virus can be easily grown on standard fibroblast cells without a requirement for complementing cell line or complex domain stabilizing SHIELD technology associated with a candidate HCMV DISC vaccine strategy [26,80]. Importantly, unlike the previously successful GPCMV DISC vaccine approach based on a capsid gene mutant, the GP83dPC+ vaccine strain did not encode the tegument protein GP83, which suggests that the immune response to the pp65 homolog antigen is not required to protect against congenital CMV, or has limited efficacy, which was predicted in our recent AdGP83 vaccine studies [28,68] Consequently, it would be worth future evaluation of the protective efficacy of a GPCMV DISC vaccine strategy lacking GP83.

Recently, we demonstrated that a recombinant Ad vector vaccine strategy encoding GP83, despite inducing a robust cell-mediated response, poorly protected against wild type challenge virus dissemination compared to an Ad gB vaccine approach [28,68]. Impaired protection was also demonstrated in the NHP model with RhCMV, where a pp65 homolog vaccine strategy against RhCMV pp65b induced a T-cell response but failed to prevent horizontal RhCMV infection [81]. Overall, this potentially indicates a general limitation of a pp65 stand-alone vaccine approach. In GPCMV, a combination vaccine strategy of GP83 and truncated gB only marginally increased protection compared to a gB only approach [69]. Importantly, the GP83 antigen behaves poorly as a cross strain protective vaccine antigen against a novel clinical strain of GPCMV, despite 100% identity in protein sequence [68]. In HCMV, a T-cell response to pp65 potentially contributes to immunosenescence long term because of the dominant T-cell response. An evaluation of the impact of the GP83 immune response in guinea pigs and immunoscence is beyond the current abilities with this animal model. However, pp65-based CMV vaccine strategies have undergone clinical trials against HCMV in transplant patients either as a standalone vaccine or in combination with gB in the context of a DNA plasmid vaccine or recombinant poxvirus-based strategies [82]. These approaches have only had moderate success in protection despite inducing a robust immune response in vaccinated patients. Potentially, the inclusion of pp65 as part of a vaccine design might also be limited if it acts as a decoy to other T-cell target antigens (e.g., IE1). Furthermore, a pp65 vaccine has the potential to additionally induce autoantibodies, but these findings were in a mouse model and might have limited correlation with humans [63]. In convalescent immunity to HCMV, the T-cell response to the IE1 protein is similar in level to that of pp65 in some HCMV infected convalescent patients [83]. Potentially, the protective immune response to IE1 is more important than that to pp65. Indeed, the IE1 response might be enhanced in a pp65 negative CMV vaccine strain compared to wild type virus. This might well be an additional factor for the success of the GP83dPC+ vaccine strategy. Importantly, an IE1 vaccine cell-mediated immune response is partially protective against horizontal virus dissemination in a RhCMV NHP model and provides better protection against horizontal CMV transmission compared to a pp65 homolog vaccine strategy [81]. We recently characterized the GPCMV major immediate early proteins (IE1 and IE2) as functional HCMV homologs [60]. In convalescent GPCMV infected animals, IE1 induces a cell-mediated response, and a recombinant defective adenovirus encoding IE1 is a more effective vaccine strategy than a recombinant AdGP83 strategy for protection against subsequent challenge with wild type virus [68] (Choi et al., paper in preparation). It is likely that the GP83dPC+ vaccine induced a cell-mediated response to IE1 similar to wild type GPCMV infected animals, and this might also contribute to protective immunity, but this awaits future evaluation for the current strategy and also the GPCMV DISC vaccine.

In this present study, GPCMV was attenuated by the deletion of the gene encoding the pp65 homolog (GP83), and this resulted in an attenuated virus with increased sensitivity to IFN-I and impaired dissemination in the animal. This current mutant virus might not be considered sufficiently attenuated for a safe live-vaccine strain in HCMV for clinical trials with a *UL83* knockout PC+ virus. Potentially, further attenuation could be applied by targeted knockout of additional pathogenicity factors such as G-protein coupled receptor (GPCR) homologs (UL33 and UL78), which are non-essential for virus growth on fibroblast cells but further attenuate virus in vivo [84,85]. However, this initial proof of principle study in GPCMV demonstrates that the T-cell response to pp65 is not absolutely required for vaccine protection against congenital CMV infection by wild type GPCMV, but an ability to cross protect between multiple strains remains to be evaluated and is an important future goal. This could be evaluated with the recent isolation of a new clinical strain of GPCMV to further raise the bar for a successful vaccine in this preclinical model [30]. Overall, the results demonstrate the high efficacy of a live virus vaccine strategy against congenital CMV infection and indicate the importance of the antibody immune response against PC in combination with monomeric and trimeric gB in contributing to protection against congenital disease in this translational model.

## 5. Conclusions

The guinea pig is the only small animal model for congenital CMV. Importantly, GPCMV encodes a functional PC for infection of non-fibroblast cells, with dissemination in vivo, including placental trophoblasts and congenital infection. The tegument protein layer of HCMV encodes a pp65 protein that is important for innate immune evasion (IFI16/cGAS-STING pathway) in the early stages of infection, but pp65 is also a T-cell target antigen and has been explored as a vaccine candidate with limited success. Earlier GPCMV studies demonstrated that GPCMV encodes a homolog pp65 protein (GP83) that is similarly involved in innate immune evasion but that a standalone GP83 vaccine provides limited protection compared to viral glycoprotein neutralizing antibody targets (gB and PC). Although the gB glycoprotein is essential for infection of all cell types, a gB immune response is insufficient to effectively neutralize infection of non-fibroblast cells, and previous gB-only-based vaccines have failed to prevent GPCMV dissemination or protection against congenital CMV above 50%. Knockout of GP83 in GPCMV attenuates the virus for infection of non-fibroblast cells and in vivo dissemination. A GP83 knockout virus was evaluated as a live attenuated vaccine strain (PC+ or PC−). The PC+ vaccine strain produced better virus neutralization on non-fibroblast cells linked to the neutralizing antibodies directed to both gB and PC. In a preconception congenital protection study, GP83dPC+ GPCMV vaccine induced sterilizing immunity and complete protection against congenital CMV infection when challenged with wild type GPCMV (strain 22,122). This demonstrated that for the guinea pig model, a fully protective vaccine against CMV requires a robust antibody response to gB and PC but that a T-cell response to the pp65 homolog is not necessary in the context of a live attenuated virus vaccine strategy. Importantly, the GP83dPC+ vaccine strategy required only a prime and single boost approach unlike a previous GPCMV DISC (PC+) vaccine that required a second booster shot to enable complete protection. However, the current live attenuated vaccine may have more limited protection against new strains of GPCMV, but this remains to be evaluated. Cross strain protection is an important hurdle in achieving an effective vaccine against congenital CMV infection, but our initial findings are encouraging.

## Figures and Tables

**Table 1:** Congenital infection outcomes.

Treatment Group (*n*)	Litter Outcomes					Outcomes for Total Pups ^a^ (no. [%])		
	Total Litters	Live Only	Dead Only	Mixed	Pre-Term	Live-Born	Still-Born	Pre-Term ^b^
GP83dPC+ (12)	12	9	0	2	1	34 [82.9]	3 [7.3]	4 [9.8]
No vaccine (14)	14	7	4	3	0	27 [56.3]	21 [43.8]	0 [0]

^a^ pup outcome between groups *p* = 0.01 (Fisher’s exact test). ^b^ dam euthanized pre-term (18 days post challenge) due to unknown illness and pups harvested.

**Table 2:** Congenital infection outcome in pups based on viral load in target organs.

Vaccine Groups (*n*)	Number [%] of Pups Positive for GPCMV in Target Organs				Number [%] of CMV + Pups ^a^
	Lung	Liver	Spleen	Brain	
GP83dPC+ (41)	0	0	0	0	0 [0]
[viral load]	[NVL] ^b^	[NVL]	[NVL]	[NVL]	
No vaccine (35)	21 [60.0]	15 [42.9]	19 [54.3]	18 [51.4]	28 [80.0]
[viral load] ^c^	[4.62 × 102]	[4.25 × 102]	[1.33 × 103]	[1.87 × 103]	

^a^*p* = 0.0001 (Fisher’s exact test), ^b^ NVL = no viral load detected, ^c^ Viral load in genome copies/mg tissue.

## Data Availability

Not applicable.

## Supplementary Materials

The following are available online at https://www.mdpi.com/article/10.3390/v13081467/s1, Figure S1. Generation of GP83 null (PC−) GPCMV mutant virus (GP83dPC−). (A) Linear diagram of the GPCMV genome showing Hind III genomic map and location of excisable BAC plasmid (GFP-BAC). Second generation GPCMV BAC is PC-negative and encodes only GP131 and GP133 and not GP12924. (B) GP83 locus encoded in HindIII ‘A’ GPCMV subgenomic fragment and location of restriction enzyme sites Bgl II and Kpn I within GP83 coding sequence used to delete majority of ORF in shuttle vector pGP83dKm (with flanking sequence in GP82 and GP84) by insertion of a kanamycin cassette by homologous recombination in place of the GP83 ORF. (C) Comparative GPCMV BAC genomic DNA restriction profile analysis (Hind III profile) of: GP83 mutant BAC (lane 1); original second generation GPCMV BAC (lane 2). Mutagenesis of the GPCMV BAC and insertion of the Km drug resistance cassette into GP83 introduced a novel Hind III restriction enzyme site creating two novel subgenomic fragments to enable verification of the modified HindIII ‘A’ locus in GP83 mutant BAC (lane 1, red dots) in comparison to original HindIII ‘A’ fragment (lane 2, yellow dot) of parental GPCMV BAC. Modification of the GP83 locus also verified by PCR and sequencing. Figure S2. Comparative virus dissemination in animals for GP83dPC+, GP83dPC− and wtGPCMV. Comparative dissemination of viruses to target organs. Three separate groups (*n* = 12 per group) of animals were infected (105 pfu, SQ) with either wt GPCMV (GP129FRT)24, GP83dPC+, or GP83dPC−, dependent upon assigned group. At various days (4, 8, 12 and 27 days post infect infection, DPI), 3 animals per group were evaluated for viral load in target organs by real time PCR of tissue extracted DNA. Viral load plotted as viral genome copies/mg tissue. Salivary gland tissue was only evaluated at day 27. Graph: A, lung; B, liver; C, spleen; D, salivary gland. Blood viremia at 4, 8, 12 and 27 DPI is shown and plotted as genome copies/mL blood (E). Statistical analysis performed by Student *t* test comparing detectable viral load in tissues or blood. * *p* < 0.05, ** *p* < 0.005.
