# Supplementary material for: A Fully Protective Congenital CMV Vaccine Requires Neutralizing Antibodies to Viral Pentamer and gB Glycoprotein Complexes but a pp65 T-Cell Response Is Not Necessary
Source: Viruses. 2021 Jul 27;13(8):1467. doi: 10.3390/v13081467 (PMC8402731; doi:10.3390/v13081467)
Supplement: Supplementary file 1 [file viruses-13-01467-s001.zip › SFigure1&2_McGregor.pptx]

## Slide 1
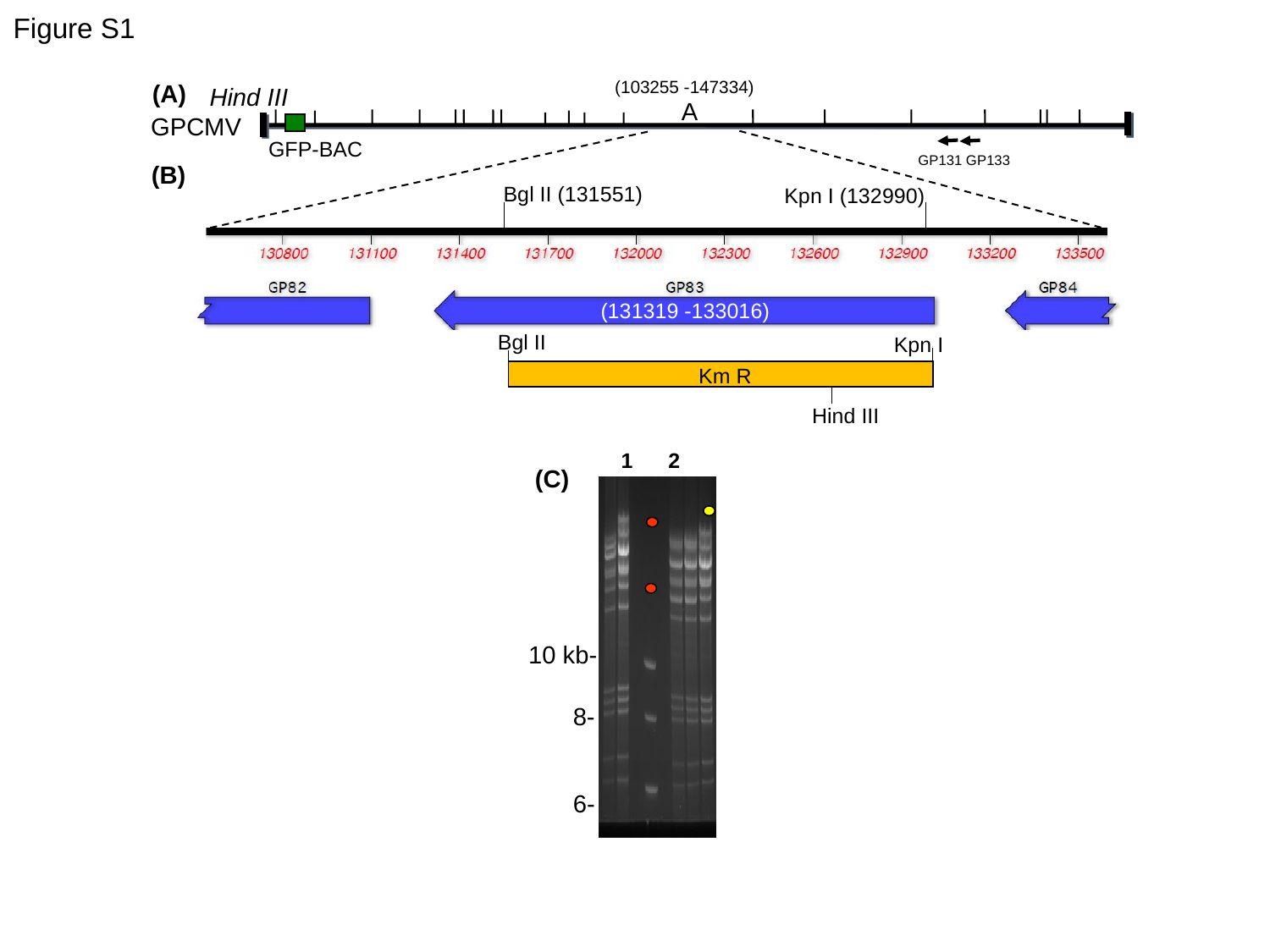

Figure S1
(103255 -147334)
(A)
Hind III
A
GPCMV
GFP-BAC
GP131 GP133
(B)
Bgl II (131551)
Kpn I (132990)
(131319 -133016)
Bgl II
Kpn I
Km R
Hind III
 1 2
(C)
10 kb-
8-
6-

## Slide 2
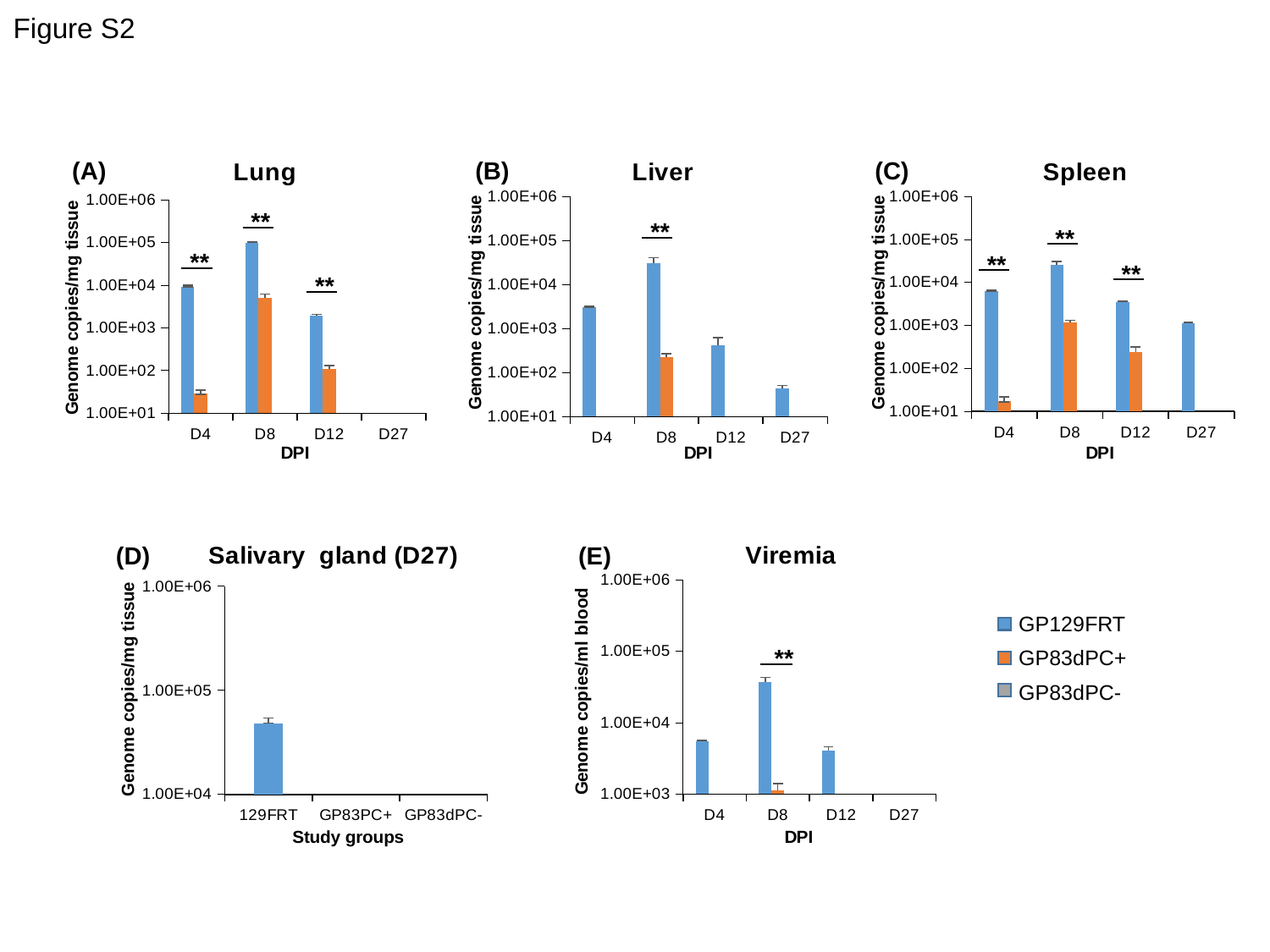

Figure S2
(A)
### Chart: Lung
| Category | 129FRT | GP83dPC+ | GP83dPC- |
|---|---|---|---|
| D4 | 9270.0 | 28.793750000000003 | None |
| D8 | 100666.66666666667 | 5020.0 | None |
| D12 | 1949.3333333333333 | 107.875 | None |
| D27 | None | None | None |**
**
**
(B)
### Chart: Liver
| Category | 129FRT | GP83dPC+ | GP83dPC- |
|---|---|---|---|
| D4 | 3033.3333333333335 | None | None |
| D8 | 30453.333333333332 | 226.75 | None |
| D12 | 422.0400000000001 | None | None |
| D27 | 44.18333333333333 | None | None |**
(C)
### Chart: Spleen
| Category | 129FRT | GP83dPC+ | GP83dPC- |
|---|---|---|---|
| D4 | 6386.666666666667 | 17.5875 | None |
| D8 | 26146.666666666668 | 1158.125 | None |
| D12 | 3530.0 | 237.01875 | None |
| D27 | 1148.3333333333333 | None | None |**
**
**
(D)
### Chart: Salivary gland (D27)
| Category | Sal gland |
|---|---|
| 129FRT | 48266.666666666664 |
| GP83PC+ | None |
| GP83dPC- | None |(E)
### Chart: Viremia
| Category | 129FRT | GP83dPC+ | GP83dPC- |
|---|---|---|---|
| D4 | 5550.0 | None | None |
| D8 | 36966.666666666664 | 1135.0 | None |
| D12 | 4025.0 | None | None |
| D27 | None | None | None |**
GP129FRT
GP83dPC+
GP83dPC-
